# Diversity of fermentative yeasts with probiotic potential isolated from Thai fermented food products

**DOI:** 10.3934/microbiol.2022037

**Published:** 2022-12-26

**Authors:** Sukrita Punyauppa-path, Pongpat Kiatprasert, Jutaporn Sawaengkaew, Polson Mahakhan, Parichat Phumkhachorn, Pongsak Rattanachaikunsopon, Pannida Khunnamwong, Nantana Srisuk

**Affiliations:** 1 Department of Mathematics and Science, Faculty of Agriculture and Technology, Rajamangala University of Technology Isan Surin Campus, Surin 32000, Thailand; 2 Department of Microbiology, Faculty of Science, Khon Kaen University, Khon Kaen 40002, Thailand; 3 Department of Biological Science, Faculty of Science Ubon Ratchathani University, Warin Chamrap District, Ubon Ratchathani 34190, Thailand; 4 Department of Microbiology, Faculty of Science, Kasetsart University, Bangkok 10900, Thailand; 5 Biodiversity Center Kasetsart University (BDCKU), Bangkok 10900, Thailand

**Keywords:** yeast probiotics, ascomycetous yeasts, phylogeny, ribosomal DNA

## Abstract

This research aimed to evaluate the diversity of yeasts recovered from fermented foods gathered from some areas of Northeastern Thailand. The fermented food items included Pla-som, Nham-pla, Kem-buknud, Isan-sausage, Pla-ra, Mhum-neu, Mhum-Khai-pla, Nham-neu, Nham-mu, Kung-joom, Som-pla-noi, and Poo-dong. Their probiotic characteristics were also investigated. A total of 103 yeast isolates of nine genera were identified using 28S rDNA sequencing. The yeast genera were *Candida* (20.3%), *Diutina* (2.9%), *Filobasidium* (1.0%), *Kazachstania* (33.0%), *Pichia* (3.9%), *Saccharomyces* (1.0%), *Starmerella* (28.2%), *Torulaspora* (2.9%), and *Yarrowia* (6.8%). Based on probiotic characteristic analysis of ten selected yeast strains, *Kazachstania bulderi* KKKS4-1 showed the strongest probiotic characteristics in terms of hemolytic activity, antimicrobial activity against pathogenic bacteria, tolerance to low pH and bile salt and hydrophobicity. Isolated yeasts with probiotic characteristics may be useful in fermented food and animal feed production to improve their nutritional values.

## 1. Introduction

Fermentation is a method of preserving excess food so it can be consumed much later without spoiling. Additionally, the ability to use microorganisms to preserve food and create flavorful fermented foods is best reflected in human culinary innovation. This is why a wide variety of fermented foods are produced, consumed and found in almost every country around the world. In some countries, these fermented foods have had an integral role in the food culture for centuries. The method of producing fermented foods is part of the wisdom and culture of each country, which has been passed down from generation to generation for hundreds of years [1].

Thailand has a tropical climate because it is close to the equator. Its warm temperature, high humidity and a relatively long rainy season cause food raw materials like meat, fish, grains, fruits, and vegetables to be highly perishable. Preservation and storage of these foods and raw materials is important to extend their shelf-life, ensuring good quality and safety for consumption, especially in rural areas. Hence, fermentation is one of the methods used to preserve food and raw materials in various communities in Thailand [2]. Fermented foods found in Thailand are diverse in terms of microbiological quality and organoleptic properties, depending on their unique ingredients and fermentation methods, which have been passed down for generations [2],[3]. Most Thai traditional fermented foods are made using simple spontaneous fermentations, which do not require expensive equipment. In spontaneous fermentation, indigenous microorganisms are derived from raw materials, utensils and the environment in combination with suitable conditions to select and promote the growth of microorganisms that cause specific fermentations in foods [3],[4].

Thai fermented foods are similar to other fermented foods because the positive organoleptic changes that occur are the result of an interaction of lactic acid bacteria (LAB) and yeasts. These two groups of microorganisms work synergistically and contribute a significant impact on taste, texture, and odor of foods [5]–[8]. However, much of the research has focused on lactic acid bacteria as the microorganisms primarily responsible for the attributes of fermented foods because they are capable of producing numerous substances such as organic acids, aromatic compounds and peptides. These substances drive fermentation processes and inhibit the growth of undesirable microorganisms. Additionally, it has been found that many of the lactic acid bacteria found in fermented foods function as probiotic bacteria [9]–[12].

Yeasts are the second most important group of microorganisms in fermented foods. They have received less attention than lactic acid bacteria. Most research has reported the presence and role of yeasts in fermented beverages rather than in fermented foods. There are few research reports on the presence of yeasts in fermented foods. These reports discuss yeasts of the genera *Candida*, *Saccharomyces*, *Lachancea*, *Pichia*, *Zygosaccharomyces*, *Debaryomyces* and *Hanseniaspora* [6],[13],[14],[15]. However, yeasts are getting more attention not only because of their flavor and aroma forming capabilities, but also for their ability to serve as probiotics. Many research articles have reported that a number of yeasts, such as *D. hansenii*, *Torulaspora delbrueckii*, *Kluyveromyces lactis*, *Yarrowia lipolytica*, *K. marxianus*, and *K. lodderae*, can tolerate gastric juices, survive in the human intestine, and are highly antagonistic to gastrointestinal pathogens [16]–[18]. Nowadays, the yeast, *S. cerevisiae* var. boulardii, is considered a probiotic strain in humans. This strain is also recommended for the prevention and treatment of various types of gastroenteritis in children and adults [19],[20].

Nowadays, consumers demand chemical-free, natural and safe foods. Fermented foods with probiotic microorganisms have become foods of choice for health-conscious people [9]. Some of these foods are not only natural and safe, but also offer health benefits. Yeast probiotics have been found to have favorable physiological effects on the human body including the synthesis of antioxidants and antimicrobials, as well as the reduction of blood cholesterol levels [5],[21],[22]. Moreover, yeasts have recently been recognized as having the potential to develop new types of probiotics [23]. They are also physiologically important in the gastrointestinal (GI) tract, although they account for a small proportion of the intestinal microflora [23],[24].

To the best of our knowledge, there are few, if any studies, on probiotic yeast diversity in fermented foods, especially in Thai fermented foods. For this reason, the current research aimed to study yeast diversity in various types of Thai fermented foods, namely fermented fish, shrimp, crab, and shellfish products and to investigate their probiotic properties. Isolated yeasts with probiotic characteristics may be useful in fermented food and animal feed production to improve their nutritional values.

## 2. Materials and methods

### 2.1. Collection of fermented food products

**Figure 1.**
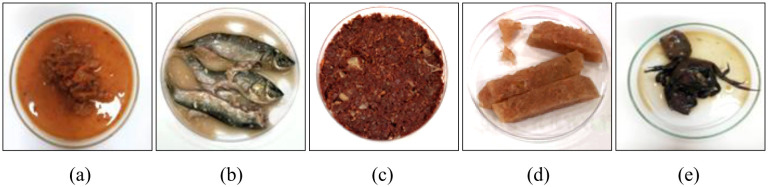
Examples of fermented food products used for yeast isolation: (a) fermented shrimp; (b) fermented fish; (c) fermented meat; (d) fermented pork; (e) fermented crab.

Thirty-eight samples (n = 38) of various fermented food such as fermented shrimp (n = 5), fish (n = 29), meat (n = 2), pork (n = 1), and crab (n = 1) were collected around the northeastern region of Thailand (Figure 1), specifically in Surin, Khon Kaen, Srisaket, and Ubon Ratchathani provinces (Table 1). All samples used in this study were purchased from local markets.

**Table 1.** List of fermented food products used for yeast isolation.

Sample code	Type of fermented food	Source	Number of yeast isolates
1, 28, 29, 30, 31	Fermented shrimp (n = 5)	Surin, Ubon Ratchathani, Khon Kaen	30
2, 3, 5, 6, 8, 9, 10, 12, 13, 14, 15, 16, 17, 18, 19, 20, 21, 22, 23, 24, 25, 26, 27, 32, 33, 34, 35, 36, 37	Fermented fish (n = 29)	Surin, Ubon Ratchathani, Khon Kaen	68
4, 7	Fermented meat (n = 2)	Ubon Ratchathani	2
11	Fermented pork (n = 1)	Ubon Ratchathani	1
38	Fermented crab (n = 1)	Khon Kaen	2
Total	n = 38		103

### 2.2. Recovery and isolation of yeasts

Yeast was isolated from each sample using the spread plate technique of Limtong et al. [25] with slight modification. One hundred microliters (µl) of an aqueous dilution were spread on yeast extract peptone dextrose (YPD) agar containing 1% yeast extract, 2% peptone, 2% glucose, and 1.5% agar supplemented with 0.25% sodium propionate. The plates were incubated at 35 °C for 48 h. The yeast colonies were picked and purified by cross streaking on the same medium. All 103 isolates of purified yeast cultures (Table 1) were preserved in YPD broth supplemented with 20% (v/v) glycerol and stored at -20 °C.

### 2.3. Characterization and identification of yeast strains

The selected yeast strains were categorized through morphological, physiological, and biochemical means following the methods of Limtong et al. [25] and Yarrow [26]. Total genomic DNA was extracted from whole yeast cells and amplified by adapting the procedures of Lachance et al. [27] and Limtong et al. [25]. Molecular identification via the D1/D2 region of the large subunit (LSU) rRNA genes and the internal transcribed spacer (ITS) region was verified using a polymerase chain reaction (PCR) technique. PCR amplification of these genes was carried out using two primer pairs: NL1-F and NL4-R [28], and ITS1 F and ITS4-R [29] as shown in Table 2. The PCR products were purified using a Qiagen Universal DNA Purification Kit (Hilden, Germany) according to the manufacturer's protocol. Purified PCR products were sequenced by Macrogen, Inc., South Korea via Gibthai Co., Ltd, Thailand. The sequences were compared using the BLASTN search program [30], assembled, and aligned using the MEGA version 7 program [31]. Phylogenetic placement of the yeast species was based on the maximum-likelihood method, applying the general time-reversible (GTR) model and used the concatenated ITS and D1/D2 sequences. Confidence levels of the clades were estimated from bootstrap analyses (1,000 replicates) [32].

**Table 2.** The primer sequences used in this study.

PPrimer names	Sequences	Specific region
NL1-F	5′-GCATATCAATAAGCGGGGAAAAG-3′	D1/D2 region of the LSU rRNA genes [28]
NL4-R	5′-GGTCCGTGTTTCAAGACGG-3′	
ITS1-F	5′-TCCGTAGGTGAACCTGCGG-3′	ITS region [29]
ITS4-R	5′-TCCTCCGCTTATTGATATGC-3′	

### 2.4. Characterization of yeast isolates for probiotic potential

#### 2.4.1. Hemolytic activity

The selected yeast strains were determined harmless using the results of a hemolysis assay. Sheep's red blood agar plates were cross-streaked with one loopful of yeast colony and incubated at 35 °C for 48 h. The types of hemolysis were determined according to appearance of zones around colonies. β-hemolysis, γ-hemolysis and non-hemolysis were designated when there were clear zones, green-hued zones and no zone around colonies, respectively.

#### 2.4.2. Antimicrobial activity

The antimicrobial activity of selected yeast strains was assessed using an agar well diffusion method according Ragavan and Das [33]. Yeast strains were cultured in YPD broth, incubated at 35 °C, and centrifuged at 4000 rpm for 5 min to separate the supernatant from the cells. The pathogenic bacteria, *Escherichia coli* TISTR 073, *Staphylococcus aureus* TISTR 029, *Enterobacter aerogenes* TISTR 1540, and *Bacillus cereus* TISTR 678, were spread on YPD plates. Then, a sterile cork borer was used to cut 0.8 mm diameter holes into the YPD agar. Aliquots of 100 µL of supernatants were placed into the wells and the plates incubated at 35 °C for 48 h. The results were obtained by measuring the width of the inhibition zones surrounding the wells.

#### 2.4.3. Acid resistance

The acid resistance test was adapted from Aloğlu et al. [34]. Selected yeasts were inoculated in YPD broth, incubated at 35 °C, and centrifuged at 4000 rpm for 5 min. After that, the pelleted cells were mixed with phosphate buffered saline (PBS) at pH 7.0 to prepare cell suspensions of approximately (log 6 CFU/ml) of the yeast cell. Acid resistance was tested by mixing a suspension of each strain with sterile PBS at pH 3.0. Before use, the pH of the PBS was initially adjusted by addition of 5N HCl and then switching to 0.1 N HCl as the solution approached pH 3.0. The suspensions were incubated at 35 °C for 0 and 3 h. The surviving microorganisms were determined via a plate count method using YPD agar. The results were reported as the %survival rate [21] calculated as:

$$\text{\%}Survival\;rate=\left(\frac{\log\frac{CFU}{mL}(t_{3})}{\log\frac{CFU}{mL}(t_{0})}\right)x100$$ (1)

Where log CFU/ml (t_0_) is the yeast cell count (log CFU/ml) at the initial time, and log CFU/ml (t_3_) is the yeast cell count (log CFU/ml) after a given incubation time.

#### 2.4.4. Bile salt tolerance

Bile salt tolerance tests were performed using the slightly modified method of Aloglu et al. [34]. A yeast cell suspension prepared, as described in the previous section, was transferred into a YPD broth containing 0.3% OX-bile salt and incubated at 35 °C. The % survival was determined from the log CFU/ml cell counts at 3 and 6 h. Equation (1) was used to determine the % survival.

#### 2.4.5. Hydrophobicity assay

Adhesion capability was determined using a modified method of Ragavan and Das [33]. Each yeast strain was enriched in YPD broth for 8 h (to an initial cell count of ~log 6 CFU/ml) and centrifuged at 4000 rpm for 5 min. After that, the cells were washed twice with PBS at pH 7.0 and re-suspended in the same buffer. Then, the cell suspension was pipetted into a 96 well plate and absorbance at 660 nm (A_1_) was measured using a microplate reader (Biorad iMark, USA). For the adhesion test, the cell suspension used to obtain the A_1_ value was mixed well with xylene and incubated for 2 h. The absorbance of the upper phase, containing the cell suspension, was measured at 660 nm (A_2_). The % adhesion was determined as:

$$\text{\%Adhesion}=(\frac{{\text{A}}_{1}-{\text{A}}_{2}}{{\text{A}}_{1}})x100$$ (2)

## 3. Results and discussion

### 3.1. Isolation and identification of yeast isolates

All 103 yeast isolates were from different fermented food products and sources (Table 1). Pure cultures of yeast isolates were classified using morphological characteristics. One of the most obvious morphological features of one yeast colony was its milky or creamy white color and circular colonies that were raised on YPD agar plate. The yeasts in this work exhibited unique morphology. The majority were ovoid or round while others were cylindrically shaped. Several studies reported that yeast shapes isolated from fermented food products varied from oval to cylindrical [35],[36].

**Figure 2.**
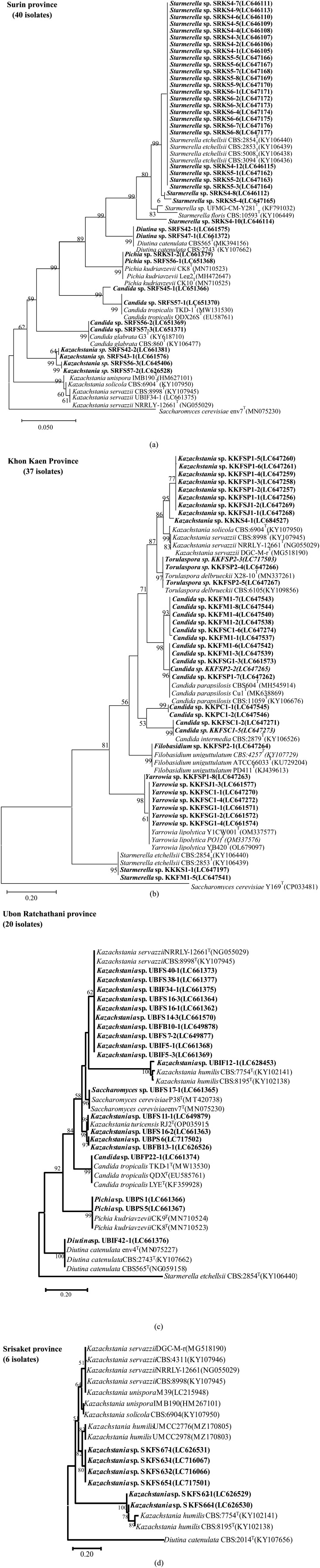
Phylogenetic tree constructed by the neighbor-joining method showing the position of strains and related yeast species based on 28S rDNA sequences and the bootstrap consensus tree inferred from 1000 replicates of the fermented food in four provinces: (a) Surin province 40 isolates; (b) Khon Kaen province 37 isolates; (c) Ubon Ratchathani province 20 isolates; (d) Srisaket province 6 isolates.

The 103 isolated yeasts were identified using 28S rDNA sequencing. They were of nine genera including *Candida* (20.3%), *Diutina* (2.9%), *Filobasidium* (1.0%), *Kazachstania* (33.0%), *Pichia* (3.9%), *Saccharomyces* (1.0%), *Starmerella* (28.2%), *Torulaspora* (2.9%), and *Yarrowia* (6.8%). These details are shown in Table 3 and the phylogenetic tree of the closest species is shown in Figure 2. The most dominant species were of the genera *Kazachstania* (33.0%) and *Starmerella* (28.2%), namely *Kazachstania* sp., *Kazachstania bulderi*, *Kazachstania exiqua, Kazachstania humili, Kazachstania servazzii, Kazachstania turicensis, Starmerella etchellsii and Starmerella roubikii*.

Most research on fermented food has focused on the lactic acid bacteria rather than yeast. So, many genera and species of lactic acid bacteria in various types of fish or meat-based fermented foods were screened, isolated, identified and reported [7],[37]–[41]. Little research has been conducted and published on yeasts in fermented foods. The most common yeasts in fermented foods are those of the genera *Candida, Saccharomyces, Pichia*, and some species in *Zygosaccharomyces, Debaryomyces* and *Hanseniaspora* [13],[15],[41]. However, our finding is the first that revealed *Kazachstania* (33.0%) and *Starmerella* (28.2%) are two dominant genera that are widely distributed in fermented food samples. Moreover, it is also the first report of *Starmerella roubikii* found in fish or meat-based fermented foods.

Yeasts of the genera *Kazachstania* and *Starmerella* are osmophilic yeasts isolated from high salt or sugar food products such as kimchi, miso, soy sauce, jams and honey [42]–[44]. Furthermore, *Kazachstania*, and *Starmerella*, provide the desirable characteristics of yeasts in food and beverage fermentations such sourdough, miso, soy sauce, chili-paste and wine [43]–[47]. The yeasts in our study possibly demonstrated the significant role in the formation of desirable colors by yeasts, textures and aromas of fish or meat-based fermented foods.

In traditional fermented foods, spontaneous fermentations often involve interactions between various microbial groups. Each of these interactions creates a group of heterogeneous microorganisms that work synergistically, producing a significant impact on taste, texture and odor [5],[8]. However, the role of *Kazachstania*, and *Starmerella* in flavor formation in fish or meat-based fermented foods remains to be determined.

**Table 3.** Yeast diversity found in the current study.

Species	Number of strains	Diversity of strains (%)
*Candida glabata*	2	1.9
*Candida intermedia*	2	1.9
*Candida metapsilosis*	7	6.8
*Candida parapsilosis*	4	3.9
*Candida prachuapensis*	1	1.0
*Candida pseudolambica*	2	1.9
*Candida tropicalis*	3	2.9
*Diutina catenulate*	3	2.9
*Filobasidium uniguttulatum*	1	1.0
*Kazachstania* sp.	11	10.7
*Kazachstania bulderi*	1	1.0
*Kazachstania exiqua*	8	7.8
*Kazachstania humili*	2	1.9
*Kazachstania servazzii*	10	9.7
*Kazachstania turicensis*	2	1.9
*Pichai kudriavzevii*	4	3.9
*Saccharomyces cerevisiae*	1	1.0
*Starmerella etchellsii*	28	27.2
*Starmerella roubikii*	1	1.0
*Torulaspora delbrueckii*	3	2.9
*Yarrowia lipolytica*	7	6.8
Total	103	100.0

### 3.2. Probiotic characteristics of yeast species

#### 3.2.1. Hemolytic activity

Several yeast strains have been reported to have hemolytic activity that can cause anemia and edema in a host [48], Hence, they are not acceptable as probiotics. Since microorganisms to be used as probiotics must be safe, they are normally tested to ensure that they are free from hemolytic activity. In this study of 103 isolated yeast strains, ten strains had no hemolytic activity suggesting that they may be safe for further use (Table 4). These ten nonhemolytic yeast strains were selected for further study of their probiotic characteristics.

#### 3.2.2. Antimicrobial activity

Of the ten nonhemolytic yeast strains, all of them except *S. etchellsii* SRKS4-12 exhibited antimicrobial activity against at least one tested pathogenic bacterial strain (Table 4). Among the antimicrobial active strains, *K. bulderi* KKKS4-1 and *K. exigua* KKFSP1-1 had the strongest antimicrobial activity, inhibiting three out of four pathogenic bacteria used in this study, including *B. cereus* TISTR 678, *E. coli* TISTR 073, and *Ent. Aerogenes* TISTR 1540. Additionally, *Y. lipolytica* KKFSC1-1, *T. delbrueckii* KKFSP2-3, *P. kudriavzevii* UBPS-1 and *S. cerevisiae* UBFS17-1 inhibited two bacterial strains while *F. uniguttulatum* KKFSP2-1*, K. servazzii* UBIF5-1 and *K. turicensis* UBFS11-1 inhibited only one bacterial strain (Table 4). The results are consistent with earlier studies that described the antibacterial activity of yeasts in the genera *Kazachstania*, *Pichai* and *Saccharomyces* [49]–[52]. However, *Starmerella* and *Torulaspora* yeasts, for which our results showed moderate inhibition against test bacteria, have been reported of having a good ability to inhibit the growth of yeasts and molds, while yeasts of the genus *Yarrowia* have not been reported to produce antimicrobial compounds [53],[54]. Yeasts with antagonistic activity against other microorganisms are considered killer yeasts. They have been reported capable of secreting proteinaceous toxins against some pathogenic bacteria, molds and yeasts [55]–[57]. The present finding is consistent with earlier research which found that nine yeast isolates were active against five different pathogens, including *Bacillus* spp, *E. coli*, *Staphylococcus*, *Pseudomonas* and *Klebsiella* [22],[33].

**Table 4.** Hemolytic and antimicrobial activities of yeast strains.

Yeast species	Hemolytic activity	Antimicrobial activity			
		*B. cereus*	*E. coli*	*Ent. Aerogenes*	*S. aureus*
*S. etchellsii* SRKS4-12	-	-	-	-	-
*K. bulderi* KKKS4-1	-	+	+	+	-
*K. exigua* KKFSP1-1	-	+	+	+	-
*Y. lipolytica* KKFSC1-1	-	+	+	-	-
*F. uniguttulatum* KKFSP2-1	-	+	-	-	-
*T. delbrueckii* KKFSP2-3	-	+	+	-	-
*K. servazzii* UBIF5-1	-	+	-	-	-
*K. turicensis* UBFS11-1	-	+	-	-	-
*P. kudriavzevii* UBPS-1	-	+	+	-	-
*S. cerevisiae* UBFS17-1	-	+	+	-	-

*Note: Zone formation (-) negative; (+) positive

#### 3.2.3. The survival rate of acid resistant and bile salt tolerant yeasts

**Figure 3.**
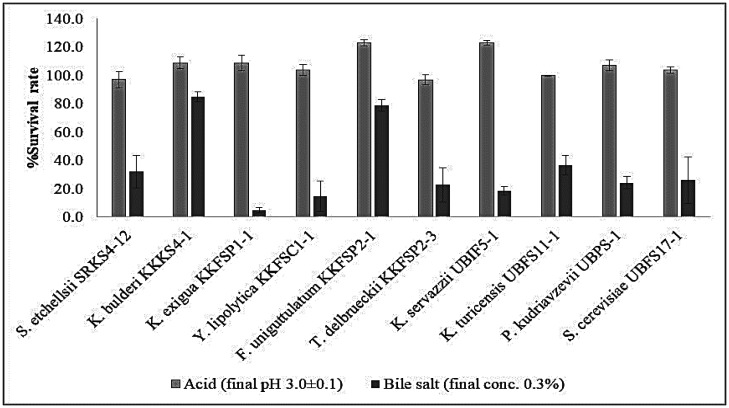
Survival rates of selected acid resistant (pH 3.0) and bile salt tolerant (0.3%) yeasts under stimulated conditions.

For probiotic microorganisms to exert their health benefits in the human body, they have to pass through extreme conditions in the gastrointestinal tract, i.e., low pH in the stomach and high bile salt concentrations in the small intestine [58],[59]. Therefore, 10 selected yeast strains with no hemolytic activity were tested for their tolerance in simulated gastrointestinal conditions. The results showed that all yeast strains were tolerant to an acidic condition at pH 3 (Figure 3). Furthermore, this acidic condition seemed to favor the growth of the yeast strains indicated by their survival rates which were above 100%. In contrast, growth of most studied yeast strains was suppressed by 0.3% bile salt (Figure 3). Bile salt has been reported to cause unfolding and aggregation of proteins in microorganisms, leading to inhibition of growth [60]. When combining both acid and bile salt resistance, *K. bulderi* KKKS4-1 and *F. uniguttulatum* KKFSP2-1 were the most tolerant strains to these harsh conditions (Figure 3). These results are consistent with those of Kourelis et al. [61], Amorim et al. [62] and Hsiung et al. [63] who reported that isolated yeast probiotics were acid tolerant at a pH of 3 and tolerant to bile salts at concentrations of 0.2–2%.

Probiotic microorganisms need to be resistant to gastric acidity and tolerant to bile salts in the gastrointestinal tract. Therefore, acidity and bile tolerance are considered essential properties required for the survival of probiotic microorganisms. However, there is no clear consensus on the exact bile concentration that probiotic microorganisms should tolerate. This is because the concentration of bile and acids in the human gastrointestinal tract varies depending on the individual, as well as the type and amount of food ingested. Normally, the mean concentration of bile salts in the small intestine is about 0.2 to 0.3% [63],[64]. It must be noted that the tolerance of yeast strains in this study may not reflect their actual ability to tolerate acid and bile salts in the human body. This is because *in vitro* evaluations do not fully mimic the actual *in situ* conditions of the human gut ecosystem. Additionally, there are other conditions and physiological factors that can affect the survival rates of yeast strains. However, the *in vitro* acid and bile salt tolerance assays used in this study remain an effective tool for rapid screening of yeast strains [63].

#### 3.2.4. Hydrophobicity assay

**Figure 4.**
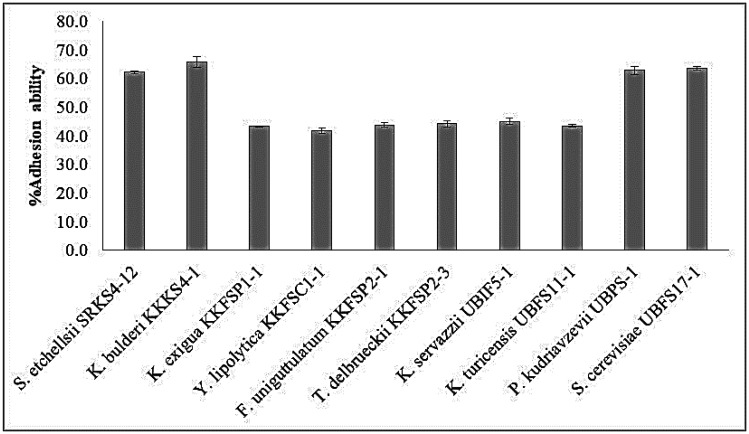
The percentage of adhesion of potential yeast strains to xylene.

The ability to adhere to mucus and epithelial cells is an important selection criterion for potential probiotic strains. Adhesion of probiotic strains is commonly studied with *in-vitro* model systems using a hydrophobicity assay. This assay determines the capability of microorganisms to adhere to surfaces having hydrophobic substances such as xylene and toluene [33]. Strains possessing high hydrophobicity exhibit adhesion and proliferation on intestinal epithelial cells [65],[66]. The experimental results revealed that most of the isolates had good hydrophobicity, ranging from 40% to nearly 70% (Figure 4). Among them, *K. bulberi* KKKS4-1, *S. cerevisiae* UBFS17-1, *P. kudriavzevii* UBPS-1, and *S. etchellsii* SRKS4-12 had the highest adhesion to xylene, 65–70% after 2 h of incubation. The overall hydrophobicity of the yeast strains in this experiment was higher than that of yeast isolated from fermented foods and beverages in Taiwan, where the values ranged from 31–63% [63]. However, several studies reported that various yeast isolates of probiotics have different hydrophobicity values. Syal and Vohra [67] reported that the yeast isolates from traditional Indian fermented foods exhibited cell surface hydrophobicity values in the range 32% to 86% with xylene and n-hexadecane. De Lima et al. [68] found that all selected *Saccharomyces cerevisiae* strains from Brazilian kefir-fermented milk had moderate to high hydrophobicity. Moreover, yeasts with high hydrophobicity have been reported. For example, *P. kudriavzevii* ROM11 isolated from cereal-based Nigerian traditional fermented food products was reported to have high adhesion to n-hexadecane [21]. Various differences in hydrophobicity of yeasts should be noted in the behaviors of yeast isolates for different hydrocarbons. As discussed above, it is difficult to simulate exact *in vivo* conditions in the laboratory. However, *in vitro* tests still provide useful information for primary screening [63].

## 4. Conclusions

This study presents yeast diversity in Thai fermented foods. Some of the isolated yeasts exhibited good probiotic characteristics including *S. etchellsii* SRKS4-12, *K. bulderi* KKKS4-1, *K. exigua* KKFSP1-1, *Y. lipolytica* KKFSC1-1, *F. uniguttulatum* KKFSP2-1, *K. servazzii* UBIF5-1, *K. turicensis* UBFS11-1, *P. kudriavzevii* UBPS-1, and *S. cerevisiae* UBFS17-1. These findings are based on population studies of yeast strains originating from meat and fermented pork and meat, or aquatic sources such as shrimp, fish, and shellfish. The current study highlights the potential of various yeast strains in the development of probiotic foods.
